# Perceived financial burden is indirectly linked to sexual well-being *via* quality of life among couples seeking medically assisted reproduction

**DOI:** 10.3389/fpsyg.2023.1063268

**Published:** 2023-04-04

**Authors:** David B. Allsop, Katherine Péloquin, Matthew T. Saxey, Meghan A. Rossi, Natalie O. Rosen

**Affiliations:** ^1^Department of Psychology and Neuroscience, Dalhousie University, Halifax, NS, Canada; ^2^Département de Psychologie, Université de Montréal, Montreal, QC, Canada; ^3^School of Family Life, Brigham Young University, Provo, UT, United States; ^4^Department of Obstetrics and Gynaecology, Dalhousie University, Halifax, NS, Canada

**Keywords:** couples, financial strain, infertility, medically assisted reproduction, personal finance, sexuality, sexual satisfaction

## Abstract

**Introduction:**

Medically assisted reproduction is a difficult treatment process for couples both financially and sexually. Yet, these two domains have not been examined together among couples seeking treatment, leaving couples and practitioners without guidance on how to address these domains together.

**Methods:**

In line with Couples and Finance Theory, we tested the hypothesis that perceived financial burden and couple income would predict quality of life during medically assisted reproduction, which would then predict four domains of sexual well-being (i.e., sexual satisfaction, desire, distress, and frequency). We also examined if the results differed by treatment status—that is, between partners who were receiving treatment and those who were not. Cross-sectional data from 120 couples who had undergone medically assisted reproduction in the past six months were analyzed *via* structural equation modeling through an actor-partner interdependence mediation model.

**Results:**

An individual’s greater perceived financial burden predicted their own lower quality of life during medically assisted reproduction, which in turn predicted their lower sexual satisfaction, desire and distress, as well as their partner’s lower sexual satisfaction. Household income did not indirectly predict any sexual well-being domains, and results regarding treatment status were inconclusive.

**Discussion:**

Clinicians can discuss with couples how perceived financial strain of medically assisted reproduction affects their quality of life and what ramifications that may have for their sexual well-being.

## Introduction

Couples seeking medically assisted reproduction (MAR) face both financial and sexual challenges. MAR is defined as “reproduction brought about through various interventions, procedures, surgeries, and technologies” (Zegers-Hochschild et al., 2017, p. 1796) and includes procedures like *in-vitro* fertilization (e.g., embryo transfer or donation), intra-uterine insemination and sperm donation, and hormonal treatments (see Passet-Wittig and Bujard, 2021). On the one hand, couples are financially burdened by the costs of MAR, which typically range between $1,197–19,840 USD per couple but can be upwards of $39,296 for those undergoing multiple cycles of *in-vitro* fertilization (Wu et al., 2014). On the other hand, couples may experience poorer sexual well-being—an evaluation of the sexual aspects of one’s life such as sexual satisfaction, desire, distress, and frequency (Dubé et al., 2020)—due to the emotional, mental, and physical strain of MAR that disrupts their intimacy (El Amiri et al., 2021). In non-MAR contexts, a growing body of literature indicates that poorer financial well-being is related to poorer sexual well-being (Wheeler and Kerpelman, 2016; Hill et al., 2017; Leavitt et al., 2019; Wikle et al., 2020; Saxey et al., 2021). Couples and Finance Theory suggests that financial processes and relationship outcomes are tied together by characteristics like life satisfaction (Archuleta and Burr, 2015). Thus, in line with this theory, the financial burden of MAR may be indirectly related to poorer sexual well-being *via* lower MAR-related quality of life. However, to our knowledge, researchers have not yet examined this notion, which leaves couples and practitioners without empirically-based guidance on how to address the impact finances may have on a couple’s quality of life and their sexual relationship.

The financial and sexual processes related to MAR can be understood through the lens of Couples and Finance Theory (Archuleta and Burr, 2015). In line with empirical evidence (for review, see Glenn et al., 2019; Dew, 2020), this theory assumes that financial processes, such as perceived financial burden from MAR and household income, are associated with the quality of a romantic relationship (Archuleta and Burr, 2015). Indeed, recent work illustrates that financial processes predict sexual satisfaction (Leavitt et al., 2019; Wikle et al., 2020; Saxey et al., 2021), sexual dissatisfaction (Hill et al., 2017), and disagreements about sex (Wheeler and Kerpelman, 2016). Couples and Finance Theory also denotes that connections between financial processes and couple relationship quality should be considered within the context of individual partner attributes such as quality of life (Archuleta and Burr, 2015). Quality of life is defined as “individuals’ perceptions of their position in life… in relation to their goals, expectations, standards and concerns” (WHOQOL Group, 1995, p. 1405). Applied to couples undergoing MAR, quality of life includes aspects such as coping, mood, and ability to communicate with one’s partner (Boivin et al., 2011). In summary, Couples and Finance Theory suggests that financial processes are associated with perceived quality of life, which will in turn be associated with the quality of a romantic relationship—including sexual well-being (Saxey et al., 2021).

### Associations between finances, quality of life, and sexual well-being

Empirical evidence supports the notion drawn from couples and finance theory that perceived financial burden and household income might be associated with MAR-related quality of life. MAR is often perceived as financially burdensome. In a study by Elliott et al. (2016), 47% of those undergoing MAR reported that paying for treatment caused financial strain. From an objective standpoint, MAR is also expensive. In Canada and the United States respectively, one cycle of *in-vitro* fertilization represents 35 and 50% of an average person’s annual disposable income (Chambers et al., 2009), and 64% of those pursuing MAR report out-of-pocket expenses exceeding $15,000 USD (Elliott et al., 2016). In line with research that suggests perceived financial burden is negatively associated with life satisfaction (Brzozowski and Spotton Visano, 2019), the financial burden of MAR may relate to poorer quality of life during MAR. That is, those who perceive more financial burden of MAR, or those who do not have high enough incomes to pay for MAR, may report poorer quality of life.

In turn, poorer quality of life during MAR may have implications for a couples’ sexual well-being. A variety of studies provide evidence that poorer quality of life during MAR is linked with poorer sexual function (Smith et al., 2015; Lo and Kok, 2016; Coward et al., 2019; El Amiri et al., 2021; Wang et al., 2022). For example, in a cross-sectional study, El Amiri et al. (2021) showed that poorer MAR-related quality of life was linked with lower sexual desire and satisfaction for both members of the couple. In addition, studies among women undergoing MAR indicate that poorer quality of life during MAR is related to greater stress (Swift et al., 2021) and poorer emotional function, such as depressive symptoms (Szigeti et al., 2020) or problems with identifying and expressing feelings (Renzi et al., 2020)—both of which are associated with poorer sexual satisfaction among those seeking MAR (Karlidere et al., 2007; Coward et al., 2019; Ho et al., 2020; Nakic Rados et al., 2020). Importantly, while links between quality of life during MAR and sexual satisfaction and sexual desire have been explored, prior studies did not examine links between quality of life during MAR and sexual distress or sexual frequency.

Couples and Finance Theory also suggests that financial and relational processes are interrelated between romantic partners (Archuleta and Burr, 2015). Thus, one person’s MAR-related financial processes and quality of life may have implications for the other partners’ sexual well-being. Indeed, an individual’s own greater perceived economic pressure and less healthy financial behaviors correlates with their partner’s lower sexual satisfaction (Saxey et al., 2021), and quality of life positively correlates among partners (Song et al., 2016). Among MAR samples, there are no studies to our knowledge that have examined finances and sexual outcomes together. However, there is evidence that an individual’s own lower sexual function predicts their own and their partner’s lower quality of life during MAR (Wang et al., 2022). Further, infertility-related personal and relational stressors, which is part of MAR-quality of life (Boivin et al., 2011; El Amiri et al., 2021), have been found to be negatively correlated with both partners’ reports of sexual function and sexual satisfaction (El Amiri et al., 2021).

### Treatment status as a moderator

It is possible that financial experiences, perceptions of quality of life, and sexual experiences during MAR may differ depending on whether an individual in a couple undergoing MAR is the one physically receiving treatment or whether they are supporting their partner who is receiving treatment. It is common for only one partner in a couple to undergo MAR (see Greenfeld and Seli, 2013; Eunice Kennedy Shriver National Institute of Child Health and Human Development, 2018), although both members of the couple are affected by the experience. However, to our knowledge, no studies have examined differences in financial, quality of life, or sexual outcomes among couples seeking MAR. Nevertheless, receiving MAR imposes a psychological and relational toll that goes beyond being childless (Greil et al., 2011; Yamanaka-Altenstein et al., 2021), and receiving MAR treatments can be painful and uncomfortable (Cousineau and Domar, 2007). Thus, it is plausible that a mediational path between an individual’s own financial predictors, own quality of life during MAR, and own sexual well-being may be stronger for treatment partners relative to support partners.^1^

### The current study

**Figure 1:**
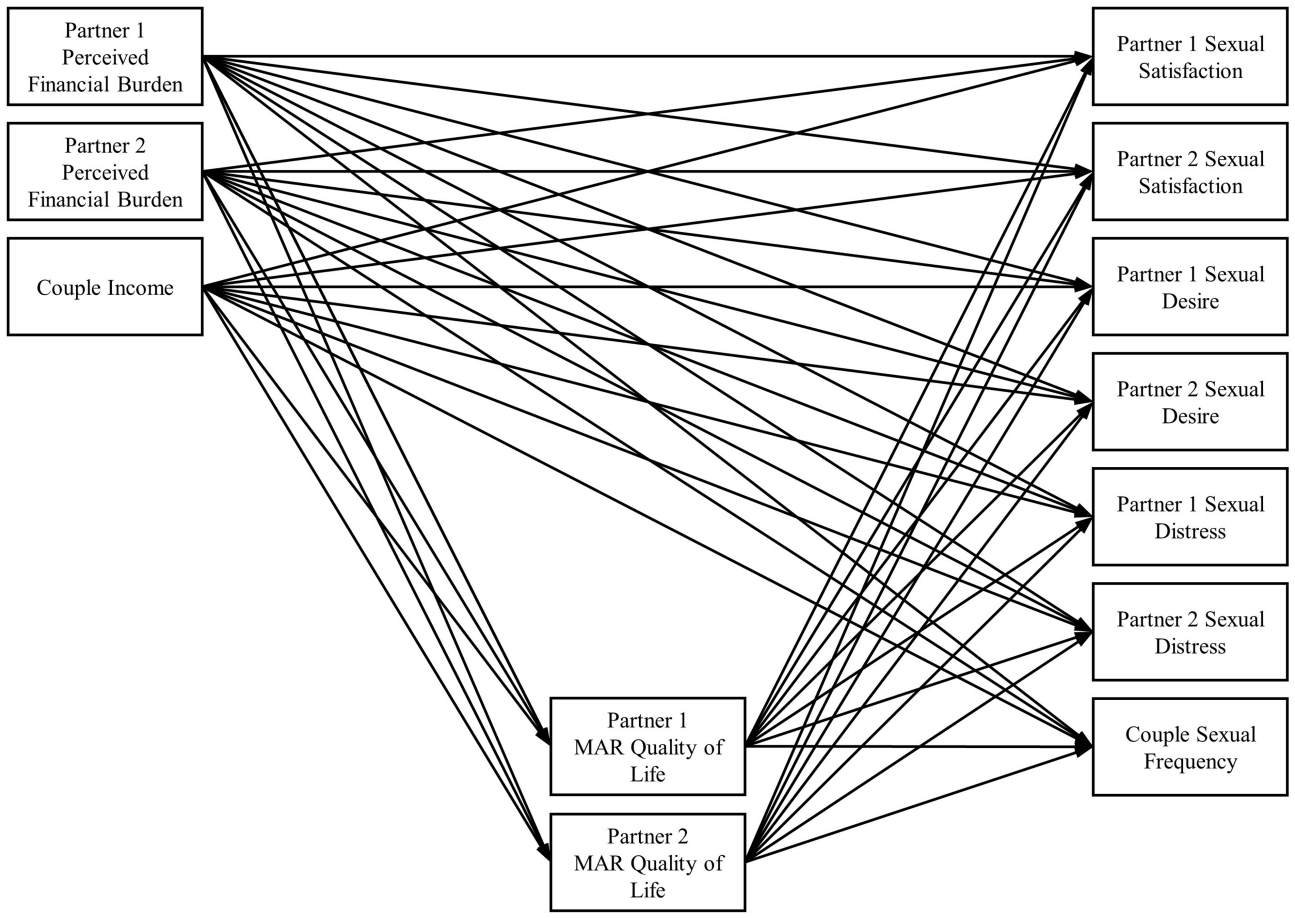
Path diagram with MAR quality of life as mediators between financial and sexual variables. MAR, medically assisted reproduction. Covariance paths not shown for parsimony.

In summary, Couples and Finance Theory suggests quality of life is an intermediary between financial and relational processes (Archuleta and Burr, 2015). Furthermore, there is evidence that poorer financial well-being, such as greater perception of financial burden and lower income levels, relates to poorer quality of life. And in turn, a body of work provides evidence that quality of life during MAR positively relates to sexual well-being. However, this evidence is preliminary given that prior literature on MAR and quality of life has almost exclusively focused on a single aspect of sexual well-being (sexual problems—specifically sexual function) rather than a variety of distinct sexual well-being domains and on the person receiving MAR, which neglects partners’ experiences. In addition, no studies exist to our knowledge that have examined finances and sexual outcomes together in a sample undergoing MAR and, by consequence, have also not considered the role quality of life during MAR may play in the association between these two constructs. This knowledge gap leaves couples undergoing MAR and the practitioners who assist them without a holistic, empirically based picture of how finances and sexual relationships—both of which are substantially impacted by MAR—are connected. Thus, the objective of this study was to examine links between finances and sexual well-being *via* quality of life during MAR. We tested two hypotheses related to a subjective (perceived financial burden) and an objective (income) financial process: (1) an individual’s greater perceived financial burden of MAR would be associated with their own and their partner’s poorer quality of life during MAR, which would in turn be associated with poorer sexual well-being for both members of the couple (i.e., lower sexual satisfaction, sexual desire, and sexual frequency, and higher sexual distress); (2) a couple’s lower household income level would be associated with poorer quality of life during MAR for individuals and their partner, which in turn would be associated with poorer sexual well-being for both members of the couple (see Figure 1 for a depiction of the conceptual model). We also hypothesized that (3) treatment status (being a treatment or support partner) would moderate the indirect path between an individual’s perceived financial burden, their quality of life during MAR, and their own and their partner’s sexual well-being outcomes such that effects will be stronger for treatment partners relative to support partners.

## Materials and methods

### Participants

Couples seeking MAR were recruited from Canada and the United States to participate in an ongoing longitudinal study examining psychological, relational, and sexual well-being during MAR. Couples must have undergone MAR in the past 6 months and not been currently pregnant to participate. If they had accessed a clinic in the past, then it must have been at least 1 year since their last appointment (the majority of couples had not undergone treatment prior to participating). Additional eligibility criteria included both members of the couple, being 18 years of age or older, fluent in English or French, living in Canada or the United States, and have neither partner self-reporting unmanaged symptoms of severe mental illness (e.g., psychosis). The final sample included 120 couples. Sociodemographic characteristics of the sample are presented in Table 1. A diagram of the flow of recruitment can be found in Supplementary Figure S1 on the Open Science Framework at https://osf.io/mc574.

**Table 1:** Sociodemographic Characteristics for the Sample (*N* = 240 individuals, 120 couples).

Variable	*M* (*SD*) (actual range)	*N* (%)
**Age (years)**	32.98 (4.93) (21–46)	
**Sex**		
Female		129 (53.8)
Male		99 (41.3)
Additional^a^		2 (0.8)
**Highest education level**		
Less than high-school		5 (2.1)
High school diploma or GED		30 (12.5)
Community college diploma		39 (16.3)
University degree		86 (35.8)
Masters/Ph.D.		49 (20.4)
Second university degree (e.g., MD, Law, MBA)		16 (6.7)
Additional^b^		6 (2.5)
**Employment status**		
Full-time employment		193 (80.4)
Part-time employment		16 (6.7)
Unemployed		11 (4.6)
Student		5 (2.1)
Additional^c^		6 (2.5)
**Couple Income** ^d^	8.74 (2.66) (3–16)	
**Ethnicity** ^e^		
White		209 (87.1)
Black		7 (2.9)
Latino/Hispanic		4 (1.7)
Asian		9 (3.8)
Middle eastern		2 (0.8)
Additional^f^		9 (3.8)
**Relationship length (years)**		
0–4		58 (24.2)
5–9		97 (40.4)
10–14		58 (24.2)
15–19		14 (5.8)
20 or more		4 (1.7)
**Relationship status**		
Married		134 (55.8)
Engaged		25 (10.4)
Common-law		72 (30)
**Country of residence**		
Canada		215 (89.6)
United States		12 (5)
**Couple relationship type**		
Same-gender		11 (9.2)
Mixed-gender		99 (82.5)

M, mean; N, number of participants; SD, standard deviation; %, percentage of sample. Percentages do not add to 100% (and counts do not add to 240 or 120) due to missing data.

a Includes two additional categories not listed to avoid identifying participants.

b Examples include professional certificate, beauty school.

c Examples include on leave, stay at home parent.

d This is the summed personal income of both partners. The range of the item individual participants responded to includes 1 (less than $15,000), 2 ($15,000–$29,999), 3 ($30,000 to $49,999), 4 ($50,000 to $69,999), 5 ($70,000 to $89,999), 6 ($90,000 to $109,999), 7 ($110,000 to $149,999), and 8 (over $150,000). As a scoring example, if one member of the couple specified their personal income ranged from $30,000 to $49,999 (score of 3) and their partner specified their personal income ranged from $50,000 to $69,999 (score of 4), the couple’s combined score would be 7.

e Participants could endorse more than one category.

f Examples include South Asian/Indian, Russian, Egyptian, blended ethnicity.

### Procedure

Recruitment occurred between November 2019 and August 2021 by research teams at Dalhousie University and Université de Montréal. Recruitment occurred both in-person at an assisted reproductive therapies clinic in the Atlantic Provinces of Canada (i.e., reviewing medical records, appointment records; 67% of sample), and through online and community advertisements posted on websites across Canada and the United States (e.g., Facebook), in local community centers, and stores, ART clinics, and other health offices (33% of sample). Participants were screened by a research assistant in-person or by phone prior to participation to confirm eligibility. Participants independently completed validated, online questionnaires, sent *via* email and hosted on Qualtrics at baseline, 6-, 12-, 18- and 24-month follow-ups for the larger study. Only data from the 6-month time point were utilized given that all measures required for the current study were available beginning at that time point and that missing data tends to increase over time in longitudinal studies (Asendorpf et al., 2014). Only data from couples undergoing treatment in the past 6 months were utilized. Participant retention strategies, such as emails, phone calls, and infographics, were used to promote participation (e.g., Rosen et al., 2020). Couples received up to $144 CDN ($57 each) in their choice of an online gift card for participating in the full study. All procedures were approved by each participating university’s Research Ethics Boards.

### Measures

#### Perceived financial burden

Perceived financial burden of MAR was assessed using a single item created for the current study in response to participant feedback on the importance of capturing the financial burden of MAR. Single item assessments of financial burden have been used successfully in prior studies (e.g., Chongpison et al., 2016). Participants received the following instructions regarding the burden of MAR, “Medically assisted reproductive services can be difficult to cope with for several reasons and may have a negative impact on several areas of your life. How did you experience the following aspects in relation to your fertility treatments? Think of your own experience not your partner’s, which may differ from yours. Check ‘does not apply’ if you have not experienced one of these aspects in your journey” and then completed several items including this one related to financial burden: “The financial burden of the treatments or the uncertainty surrounding the costs.” The item was rated on a 7-point scale where the lowest point was “1 not difficult at all,” the middle points were “2,” “3,” “4,” “5,” and “6,” and the highest point was “7 extremely difficult.” Responses marked as “Does Not Apply” (*n* = 8 participants) were recoded to equal one to reflect the notion that if financial burden of MAR was not applicable to an individual, it likewise would not be difficult. Higher scores reflect greater perceived financial burden of MAR.

#### Income

Income was assessed using a single item, “What is your annual personal income before tax deduction? Do not include your partner’s income.” The item was rated on an 8-point scale (1 = less than $15,000 to 8 = over $150,000). Both partners’ responses were summed to create a measure of couple-level household income.

#### Quality of life during MAR

Quality of life during MAR was assessed using the fertility quality of life (FertiQol) tool (Boivin et al., 2011). Participants responded to 24 items such as, “Do you feel drained or worn down because of fertility problems or your experience with MAR?.” The measure was adapted slightly to be inclusive of same gender/sex couples (e.g., adding “or your experience with MAR” to items). Items were rated on a 5-point Likert scale (0 = *completely* to 4 = *not at all*). Items were scaled and summed to create the 0 to 100 scale described by Boivin et al. (2011). Higher scores reflect better quality of life during MAR. The FertiQol has shown strong psychometric properties among couples seeking MAR (Arpin et al., 2019) and displayed good internal consistency in the current study (α partner 1 = 0.91, α partner 2 = 0.92).

#### Sexual satisfaction

Sexual satisfaction was assessed using the Global Measure of Sexual Satisfaction (GMSEX; Lawrance and Byers, 1995). Participants responded to five items about the quality of their overall sexual relationship on a 7-point, bipolar scale regarding five pairs of words (e.g., “very bad” and “very good”). Items were summed to create a total score and higher scores reflect greater sexual satisfaction. The GMSEX has shown strong psychometric properties among couples seeking MAR (Arpin et al., 2019) and has been validated for use among men and women (Mark et al., 2014). The scale displayed good internal consistency in the current study (α partner 1 = 0.94, α partner 2 = 0.93).

#### Sexual desire

Similar to prior studies (Schwenck et al., 2020; Rossi et al., 2022), sexual desire was assessed using two equivalent sexual desire items from either the International Index of Erectile Function (IIEF; Rosen et al., 1997) for men (i.e., “Over the past 4 weeks, how often have you felt sexual desire?”; “Over the past 4 weeks, how would you rate your level (degree) of sexual desire?”) or the Female Sexual Functioning Index (FSFI; Rosen et al., 2000) for women (i.e., “Over the past 4 weeks, how often did you feel sexual desire or interest?”; “Over the past 4 weeks, how would you rate your level (degree) of sexual desire or interest?”). Items were rated on 5-point Likert scales with 1 = low sexual desire and 5 = high sexual desire. Items were summed to create total scores, and higher scores reflect higher sexual desire. Both the IIEF and FSFI have shown strong psychometric properties generally (Rosen et al., 1997, 2000), among couples seeking MAR (El Amiri et al., 2021), and among clinical samples such as those in the transition to parenthood (Rossi et al., 2022). The two items from the IIEF (α = 0.84) and the FSFI (α = 0.91) displayed good internal consistency in the current study.

#### Sexual distress

Sexual distress was assessed using the Sexual Distress Scale – Short Form (SDS-SF; Santos-Iglesias et al., 2020), which is validated for use with both men and women. Participants responded to five items about their experience of negative emotions related to their sexual relationship (e.g., “distressed about your sex life”). Items were rated on a 5-point Likert scale (1 = *never* to 5 = *always*). Items were summed to create a total score, and higher scores reflect greater sexual distress. The SDS-SF has been validated for use among clinical samples (Santos-Iglesias et al., 2020) and has shown strong psychometric properties among non-MAR samples such as individuals affected by the COVID-19 pandemic (Gauvin et al., 2022). The scale displayed good internal consistency in the current study (α partner 1 = 0.93, α partner 2 = 0.92).

#### Sexual frequency

As *in prior* research (e.g., Rosen et al., 2020), sexual frequency was assessed using a single item, “During the past 4 weeks, how often did you and your partner engage in any sexual activity defined as oral sex, manual stimulation (touching genitals), intercourse with vaginal penetration, intercourse with anal penetration.” The item was rated on a 7-point Likert scale (0 = *not at all* to 6 = *more than once a day*). Higher scores reflect more frequent sexual activity. Given the high correlation between partner’s scores (*r* = 0.68, *p* < 0.001), scores were averaged to create a couple-level sexual frequency variable.

### Data analysis

Participant roles (i.e., who is “Partner 1” and who is “Partner 2”) were randomized prior to analyses (Kenny et al., 2006; Olsen and Kenny, 2006) as dyads were treated as indistinguishable to retain mixed-gender (*n* = 99) and same-gender (*n* = 11) couples as well as dyads where both partners were undergoing MAR (*n* = 2). Correlations were prepared in R (Version 4.2.1; R Core Team, 2022) and are presented in Table 2. We constructed an actor-partner interdependence mediation model (Ledermann et al., 2011) in Mplus (Version 8.6; Muthén and Muthen, 1998–2017) to test our hypotheses, as detailed in Figure 1. Predictors (“X” variables; see Hayes, 2018) included both partners’ perceived financial burden of MAR and couples’ household income, mediators (“M” variables) included both partners’ quality of life during MAR, and outcomes (“Y” variables) included both partners’ reports of sexual satisfaction, desire, and distress and couples’ sexual frequency. The model was run across 5,000 bootstrap samples to estimate accurate standard errors of indirect effects (Hayes, 2018). Missing data were handled *via* full information maximum likelihood, and two auxiliary variables (i.e., education levels of both partners) were included in the model to help estimate missing data (Collins et al., 2001). There were little missing data overall (83 to 92% of data present across all variables). The model fit the data well (*χ*^2^ (42) = 39.156, *p* = 0.597; CFI = 1.00; RMSEA = 0.074; SRMR = 0.072) (Hair et al., 2010).

**Table 2:** Means, standard deviations, and correlations.

Variable	*M*	*SD*	1	2	3	4	5	6	7	8	9	10	11
1. Couple income	8.74	2.66											
2. Financial burden P1	4.56	1.98	0.05										
3. Financial burden P2	4.33	2.04	−0.02	0.40^**^									
4. MAR quality of life P1	66.42	17.76	−0.04	−0.07	−0.10								
5. MAR quality of life P2	65.87	17.58	0.04	−0.08	−0.30^**^	0.06							
6. Sexual satisfaction P1	24.68	7.52	−0.07	−0.14	−0.19	0.33^**^	0.22^*^						
7. Sexual satisfaction P2	25.53	7.10	−0.13	−0.10	−0.11	0.16	0.31^**^	0.60^**^					
8. Sexual distress P1	6.56	5.05	−0.07	0.19	0.18	−0.47^**^	−0.14	−0.63^**^	−0.42^**^				
9. Sexual distress P2	6.09	4.82	0.02	0.17	0.11	−0.12	−0.48^**^	−0.48^**^	−0.65^**^	0.33^**^			
10. Sexual desire P1	7.04	2.37	0.02	−0.11	−0.02	0.40^**^	−0.12	0.37^**^	0.30^**^	−0.57^**^	−0.11		
11. Sexual desire P2	6.62	2.35	−0.35^**^	−0.10	−0.21^*^	0.07	0.29^**^	0.40^**^	0.55^**^	−0.15	−0.60^**^	0.01	
12. Couple sexual frequency	1.93	1.05	−0.19	−0.22^*^	−0.13	0.14	0.05	0.58^**^	0.53^**^	−0.39^**^	−0.33^**^	0.41^**^	0.41^**^

*Indicates *p* < 0.05; **indicates *p* < 0.01. *M* and *SD* are used to represent mean and standard deviation, respectively. P1, partner 1; P2, partner 2. Partner 1 and partner 2 roles assigned randomly. Parameters presented here are raw coefficients and were not constrained to be the same between both partners.

Moderation by treatment status was tested by running the model again but treating dyads as distinguishable based on which partner was receiving treatment (the treatment partner) and which partner was not (the support partner). Couples were removed from this moderation analysis if both partners were undergoing MAR (*n* = 2 couples) or did not provide information about the treatments they were undergoing (*n* = 7). This removal resulted in a sub-sample of 111 couples drawn from the 120 couples analyzed in the primary analysis. Treatment partners identified as women (99.1%) and men (0.9%). Support partners identified as women (11.5%), men (87.5%), and non-binary (1.0%). The model did not successfully converge when using maximum-likelihood as the estimator. Therefore, we used a Bayesian estimator (see Muthén, 2010), and the model converged. Formal tests of whether parameters differed between treatment and support partners were performed using the “Model Test” command in Mplus (Muthén and Muthen, 1998–2017). This command tested whether the indirect paths significantly differed (and not just from zero) between treatment and support partners *via* Wald Chi-square tests (Muthén and Muthen, 1998–2017). The model adequately fit the data (CFI = 0.980, RMSEA = 0.064, PPP = 0.273) (Asparouhov and Muthén, 2017)^3^.

Data, input, and output files of these models as well as full details on study measures have been posted to the Open Science Framework at https://osf.io/mc574/ to promote transparency and replicability in our analyses.

## Results

As seen in Table 3, our first hypothesis was partially supported and provided evidence of indirect effects. An individual’s greater perceived financial burden of MAR was significantly associated with their own lower quality of life during MAR and, in turn, their own lower sexual satisfaction (*B* = −0.18, 95% CI [−0.40, −0.02]), lower sexual desire (*B* = −0.07, 95% CI [−0.15, −0.01]), and higher sexual distress (*B* = 0.19, 95% CI [0.01, 0.38]). In addition, an individual’s greater perceived financial burden was indirectly associated with their partner’s lower sexual satisfaction (*B* = −0.10, 95% CI [−0.26, −0.01]) *via* the individual’s own lower quality of life during MAR. Hypothesis two was not supported as there were no significant indirect effects between income and sexual well-being variables *via* MAR-related quality of life (see Table 3).

**Table 3:** Indirect effects from primary mediation model.

Outcome	Mediator	Predictor	Estimate	Lower 95% CI	Upper 95% CI
Sexual satisfaction P1	Mar quality of life P1	Couple income	0.01	−0.09	0.12
	Mar quality of life P2		0.01	−0.05	0.08
	Mar quality of life P1	Financial burden P1	**−0.18**	**−0.40**	**−0.02**
	Mar quality of life P2		−0.01	−0.14	0.09
	Mar quality of life P1	Financial burden P2	−0.02	−0.21	0.16
	Mar quality of life P2		**−0.10**	**−0.26**	**−0.01**
Sexual satisfaction P2	Mar quality of life P1	Couple income	0.01	−0.05	0.08
	Mar quality of life P2		0.01	−0.09	0.12
	Mar quality of life P1	Financial burden P1	−0.10	−0.26	−0.01
	Mar quality of life P2		−0.02	−0.21	0.16
	Mar quality of life P1	Financial burden P2	−0.01	−0.14	0.09
	Mar quality of life P2		**−0.18**	**−0.40**	**−0.02**
Sexual distress P1	Mar quality of life P1	Couple income	−0.02	−0.13	0.09
	Mar quality of life P2		0.00	−0.04	0.01
	Mar quality of life P1	Financial burden P1	**0.19**	**0.01**	**0.38**
	Mar quality of life P2		0.00	−0.03	0.06
	Mar quality of life P1	Financial burden P2	0.03	−0.17	0.21
	Mar quality of life P2		0.03	0.00	0.12
Sexual distress P2	Mar quality of life P1	Couple income	0.00	−0.04	0.01
	Mar quality of life P2		−0.02	−0.13	0.09
	Mar quality of life P1	Financial burden P1	0.03	0.00	0.12
	Mar quality of life P2		0.03	−0.17	0.21
	Mar quality of life P1	Financial burden P2	0.00	−0.03	0.06
	Mar quality of life P2		**0.19**	**0.01**	**0.38**
Sexual desire P1	Mar quality of life P1	Couple income	0.01	−0.03	0.05
	Mar quality of life P2		0.00	−0.02	0.01
	Mar quality of life P1	Financial burden P1	**−0.07**	**−0.15**	**−0.01**
	Mar quality of life P2		0.00	−0.01	0.03
	Mar quality of life P1	Financial burden P2	−0.01	−0.08	0.06
	Mar quality of life P2		0.01	−0.01	0.06
Sexual desire P2	Mar quality of life P1	Couple income	0.00	−0.02	0.01
	Mar quality of life P2		0.01	−0.03	0.05
	Mar quality of life P1	Financial burden P1	0.01	−0.01	0.06
	Mar quality of life P2		−0.01	−0.08	0.06
	Mar quality of life P1	Financial burden P2	0.00	−0.01	0.03
	Mar quality of life P2		**−0.07**	**−0.15**	**−0.01**
Couple sexual frequency	Mar quality of life P1	Couple income	0.00	0.00	0.01
	Mar quality of life P2		0.00	0.00	0.01
	Mar quality of life P1	Financial burden P1	−0.01	−0.03	0.00
	Mar quality of life P2		0.00	−0.01	0.00
	Mar quality of life P1	Financial burden P2	0.00	−0.01	0.00
	Mar quality of life P2		−0.01	−0.03	0.00

Bolded coefficients significant at *p* < 0.05. CI, confidence interval; P1, partner 1; P2, partner 2. The P1 and P2 specifiers are arbitrary as dyads were treated as indistinguishable and roles were randomized.

With regard to our third hypothesis, we found no evidence of moderation by treatment status. Of note, treatment partner’s—but not support partner’s—greater perceived financial burden of MAR was significantly associated with their own lower sexual satisfaction (*B* = −0.37, 95% CI [−0.83, −0.08]) and higher sexual distress (*B* = 0.28, 95% CI [0.07, 0.62]). However, Wald chi-square tests indicated that the treatment and support partner indirect paths did not significantly differ from each other in terms of the indirect path related to sexual satisfaction (χ^2^(1) = 2.435, *p* = 0.119) nor the indirect path related to sexual distress (*χ*^2^(1) = 1.205, *p* = 0.272). Therefore, results regarding moderation by treatment status were deemed inconclusive.

## Discussion

This is the first study, to our knowledge, to examine a theoretically-grounded model of the associations among financial, quality of life, and sexual outcomes for couples receiving MAR. We provide evidence of an indirect pathway whereby an individual’s perception of greater financial burden of MAR relates to their lower quality of life during MAR, which, in turn, is linked to their own poorer sexual satisfaction, sexual desire, and sexual distress, as well as their partner’s poorer sexual satisfaction. Contrary to our expectations, we did not find evidence of an indirect pathway involving objective levels of household income, quality of life during MAR, and sexual well-being, nor did we find that treatment status moderated any of the effects.

Perceived financial burden of MAR indirectly predicted poorer sexual well-being across three domains—sexual satisfaction, desire, and distress (but not couples’ sexual frequency)—*via* lower MAR-related quality of life. Couples face enormous financial challenges when undergoing MAR (see Wu et al., 2014). Our results provide evidence that the more these financial challenges are perceived as burdensome, the lower the quality of life an individual has during MAR, potentially regardless of whether they are the one receiving treatment or not. This evidence is in line with research that suggests financial burden is associated with MAR-related stress (see Woods et al., 2022), which may relate to poorer quality of life during MAR. Poorer quality of life during MAR as a result of perceived financial burden may entail aspects such as poorer ability to cope with stress, physical and emotional fatigue, mood changes, and relationship challenges (Boivin et al., 2011). These effects may be especially prevalent among couples who are uncertain if they can afford future treatment and feel financial pressure for a current treatment cycle to be successful (Gameiro et al., 2012).

That three sexual domains—satisfaction, desire and distress—were indirectly predicted *via* quality of life during MAR suggests the implications of the current study are widespread for couples’ sexual well-being given that research and theory indicate that these sexual well-being facets are distinct in individuals’ lives (e.g., Dubé et al., 2020). For sexual satisfaction, we also found evidence of a dyadic process whereby one’s own greater perceived financial burden was indirectly associated with one’s partner’s lower sexual satisfaction through the individual’s lower quality of life during MAR. In non-MAR contexts, quality of life is strongly correlated with happiness (Medvedev and Landhuis, 2018), and those who feel happiest tend to also be more satisfied with their sexual relationships (see Laumann et al., 2006; Impett et al., 2014). If a partner notices their companion is unhappy, especially in terms of their romantic relationship (Fisher et al., 2015), they may feel less connected and evaluate their sexual relationship more negatively. This result aligns with prior work that suggests an individual’s financial outcomes and sexual satisfaction (Saxey et al., 2021) and general quality of life (Song et al., 2016) tend to be correlated with those of their partner. Thus, there may be a spillover effect for couples undergoing MAR such that when quality of life deteriorates as a product of financial burden, general happiness and satisfaction decline, as does sexual satisfaction for both members of the couple.

An alternative process may explain the indirect pathway for sexual desire. Toates (2009) noted the central role that contextual factors play in affecting desire to engage sexually with a partner. It may be that poorer quality of life during MAR, as a result of increased financial burden, provides an overall negative life context that includes more stress (Swift et al., 2021) and poorer psychological function (Renzi et al., 2020; Szigeti et al., 2020). According to Toates (2009), this negative context then lessens interest in and motivation for sex.

Finally, with regard to sexual distress, research among non-MAR clinical samples indicates that low levels of sexual desire (Derogatis et al., 2008) and sexual satisfaction (Bois et al., 2016) are related to greater sexual distress. One possibility is that there is a sequential pattern to these effects such that greater perceived financial burden relates to poor quality of life during MAR, which in turn relates to lower sexual desire and satisfaction, and finally results in greater sexual distress. Indeed, sexual distress is a diagnostic criterion for sexual dysfunction (American Psychiatric Association, 2013), suggesting that sexual distress is often a consequence of other sexual processes. Just as stress often follows changes in one’s circumstances (Patterson, 2002), sexual distress may follow changes in sexual satisfaction and sexual desire levels. Given the exploratory nature of the current study, this multi-mediator hypothesis was not tested post-hoc. However, future research could explore this notion.

In contrast to the findings for sexual satisfaction, we did not observe partner effects for sexual desire or distress. These results could be due to sexual satisfaction being conceptualized as a more interpersonal process because it is an evaluation of the sexual relationship (see Lawrance and Byers, 1995; Stephenson and Meston, 2010) whereas sexual desire and distress, while influenced by interpersonal factors, are more intraindividual (see Stephenson and Meston, 2010; van Anders et al., 2021). Also contrary to our hypothesis, perceived financial burden was not indirectly associated with couple sexual frequency through quality of life during MAR. Mixed-sex couples’ sexual frequency levels are often regimented by a treatment program during MAR, rather than by the couple alone, to achieve fertility goals (Stanford et al., 2002; Lundin and Elmerstig, 2015). Further, health challenges such as pain due to treatment (see Cousineau and Domar, 2007) or the side effects of medication (see Derman and Adashi, 1994) may disrupt mixed- and same-sex couples’ sexual frequency be lessening their ability and/or desire to engage in sex. Given that all couples in our sample were actively undergoing MAR, this regimentation and possible health challenges may have resulted in sexual frequency that correlated with a couple’s treatment program rather than couple-specific processes such as levels of financial burden and quality of life. And because these couple-specific processes may not have covaried with couple sexual frequency, no indirect associations were observed.

We did not find that household income was indirectly associated with sexual well-being through quality of life during MAR. Given the relatively affluent nature of the current sample (average household income ranged between $100,000 and $140,000) and that individual’s subjective evaluations of income tend to poorly match their actual income (Grable et al., 2012), it is possible that there was an incongruence between income and perceived financial burden for the couples in the current study. Specifically, the couples in the current sample may have been able to afford MAR yet still perceived it as financially burdensome. And, in line with stress theories that emphasize the role of perceptions (e.g., Patterson, 2002), perceptions of finances, rather than concrete levels of income, were linked to quality of life and sexual outcomes. Future studies among both low- and high-income couples seeking MAR may help confirm this line of thinking and increase diversity and inclusivity in MAR research. Nevertheless, subjective evaluations of financial burden—rather than more impartial financial assessments like income—may be a novel target for clinical interventions aimed at improving sexual well-being among those seeking MAR.

Finally, results regarding treatment status as a moderator of the indirect association between financial processes, quality of life during MAR, and sexual well-being were inconclusive. This result may indicate that treatment and support partners are affected similarly by the process involving finances, quality of life, and sexual well-being. This result was surprising given that MAR treatment itself imposes a significant psychological and relational toll above and beyond childlessness alone (Greil et al., 2011; Yamanaka-Altenstein et al., 2021). One possible explanation for this lack of differences between treatment and support partners in how financial variables are linked with sexual well-being through quality of life during MAR is that both partners may be empathetic toward one another and are highly involved in and affected by the MAR process. As partners show empathy toward one another and, indeed, personally share in one another’s experiences (see Wearne et al., 2019), both support partners’ and treatment partners’ experiences with MAR may come to more closely resemble one another. Indeed, prior work suggests some couples are heavily invested in bonding and being close with their partner during MAR (Sauve et al., 2020), which supports the idea that treatment is a couple experience and not just the “problem” of one partner. As well, partners likely decide to share the financial burden of MAR together given the high costs of MAR (Wu et al., 2014) and the relevance of MAR to both members of the couple.

### Strengths and limitations

One notable strength of the current study is its dyadic approach. Prior work studying MAR in general has largely included data from only one partner of a couple (e.g., Greil et al., 2011; Mosalanejad et al., 2012; Elliott et al., 2016; Swift et al., 2021); our study builds on prior work by including data from both members of a couple when examining variables that are relevant for both members of the couple. Additional strengths of the study include examining multiple domains of sexual well-being and that the sample was inclusive of both mixed- and same-gender couples.

The study has several important limitations to note. First, the study was cross-sectional. Thus, inferences about the directionality of mediation paths should be interpreted with caution. Next, the sample was primarily White, relatively affluent, and predominantly consisted of mixed-sex couples, which limits the generalizability of findings. Also, our sample was composed of only two couples where both partners were undergoing treatment, thus limiting the generalizability of findings to dual-treatment couples. It may be that the financial burden of MAR exerts an even stronger influence on quality of life and sexual well-being for dual-treatment couples as they face a potentially more stressful and more demanding course of treatment than couples where a single partner is receiving treatment (see Greil et al., 2011; Yamanaka-Altenstein et al., 2021). Further, our measure of perceived financial burden was based on a single item. A more comprehensive measure that assesses other facets of financial burden (e.g., financial sacrifices to afford MAR, congruence between MAR financial costs and personal values, financial costs of treatment) could be employed in future studies. This approach would allow researchers to understand better whether the effects of financial burden on quality of life during MAR and sexual well-being change depending on the meanings and motivations surrounding the financial burdens of MAR. As well, our perceived financial burden item may have conflated experienced financial burden with uncertainty around future financial costs—separating these two dimensions would be beneficial in future work to examine their unique impacts. Additionally, we were unable to examine gender identity as a moderator because of issues related to model fit and model convergence and because we did not want to exclude same-gender/sex couples and couples in which there are non-binary individuals from the analysis. Data on other aspects of gender such as norms and stereotypes were not collected in the current study. Future work can examine these two constructs in the context of finances, MAR quality of life, and sexual well-being in line with prior work, which suggests they are important factors in financial processes and relationship outcomes (LeBaron et al., 2018). Likewise, we could not examine associations between financial burden and the duration of treatment for couples (e.g., years of treatment, number of treatment cycles) given these variables are not available in our data and the majority of couples were undergoing treatment for the first time. Thus our sample was not well-suited to questions about treatment duration (i.e., little variance in treatment duration). Accordingly, future research might include these two variables to help clinicians potentially tailor their care around these constructs.

### Implications and conclusions

In line with Couples and Finance Theory (Archuleta and Burr, 2015), clinicians might assist couples undergoing MAR in addressing how the perceived financial strain of MAR may be affecting their quality of life, with benefits to their sexual well-being. For example, when gathering information on factors affecting sexual difficulties, clinicians should invite couples to share how finances may be affecting their quality of life and potential ramifications for their sexual well-being. When gathering information on the role of finances, therapists should also ask both members of a couple to what extent they perceive that finances are affecting their own and their partner’s quality of life, including aspects such as coping, stress, fatigue, mood, or ability to communicate. Therapists might explain that finances affect couples’ relationships more broadly (Hill et al., 2017; Leavitt et al., 2019; Wikle et al., 2020; Saxey et al., 2021) to provide context to the discussion and then inquire about potential implications for sexual satisfaction, sexual distress, and sexual desire. Cognitive-behavioral strategies—which emphasize addressing unhelpful cognitive patterns and promoting coping skills (Hofmann et al., 2013)—have been found to be useful for helping individuals manage the interference of MAR to their lives (Gorayeb et al., 2012; Mosalanejad et al., 2012). Such approaches could be applied to the domains of finances and sexual well-being during MAR.

In conclusion, this study broadens Couples and Finance Theory (Archuleta and Burr, 2015) by applying the theory to a specific clinical population—couples receiving MAR—who face both substantial expenses of MAR (see Wu et al., 2014) and challenges to their relationship quality (El Amiri et al., 2021; Yamanaka-Altenstein et al., 2021). This study was the first we are aware of to extend the theory to sexual relationships in this relevant population and provides evidence that finances and sex are linked among couples undergoing MAR through disruptions to their MAR-related quality of life.

## Data availability statement

The original contributions presented in the study are publicly available. This data can be found here: https://osf.io/mc574/.

## Ethics statement

The studies involving human participants were reviewed and approved by Dalhousie University and Université de Montréal. The patients/participants provided their written informed consent to participate in this study.

## Author contributions

DA, KP, MS, MR, and NR: conceptualization and methodology. DA: software and writing – original draft. KP, MR, and NR: investigation, resources, data curation, project administration, and funding acquisition. KP, MS, MR, and NR: writing – review and editing. DA, KP, MR, and NR: supervision. All authors contributed to the article and approved the submitted version.

## Funding

This study was funded by a grant from the Canadian Institutes of Health Research (Grant #PJT-162196) and an IWK Atlee Endowment Fund Research Grant.

## Conflict of interest

The authors declare that the research was conducted in the absence of any commercial or financial relationships that could be construed as a potential conflict of interest.

## Publisher’s note

All claims expressed in this article are solely those of the authors and do not necessarily represent those of their affiliated organizations, or those of the publisher, the editors and the reviewers. Any product that may be evaluated in this article, or claim that may be made by its manufacturer, is not guaranteed or endorsed by the publisher.

## Footnotes

^1^Given that both partners bear the financial burden of treatment, regardless of which partner receives it, we would not expect to see differences between treatment and support partners in paths from financial predictors to quality of life during MAR. However, in line with research which suggests treatment partners face an additional burden over support partners, we would expect paths from quality of life during MAR to sexual well-being to differ between treatment and support partners. Because mediational paths are a product of two other paths—(a) the path from a predictor (finances) to a mediator (MAR-quality of life) and (b) the path from a mediator (quality of life) to an outcome (sexual well-being)—if any of the two pathways differ by a moderator, the entire mediational pathway would similarly differ by that same moderator. Because we expect the second path to differ between treatment and support partners, we expect the overall mediational path to differ between partners. This expectation, and that our primary hypotheses related to mediation (see current study), led us to similarly focus our moderation hypothesis on how mediational paths—rather than direct paths from quality of life to sexual well-being—would be moderated by treatment status.

^3^We had initially planned to test whether gender was a moderator of indirect effects between finances, quality of life during MAR, and sexual well-being. We attempted this analysis by running an indistinguishable model and testing gender differences using moderation by gender. The models either did not fit the data well or did not converge in this indistinguishable model, using both maximum-likelihood and Bayesian estimation. We could have run the model by treating dyads as distinguishable, but this approach would have excluded same-gender/sex couples and couples in which there are non-binary individuals, and we did not have any theoretical or empirical reason to do so. Accordingly, we proceeded to test only treatment status as a moderator to be inclusive of same- and mixed-gender couples.
